# [Corrigendum] miR-335 promotes ferroptosis by targeting ferritin heavy chain 1 in *in vivo* and *in vitro* models of Parkinson's disease

**DOI:** 10.3892/ijmm.2025.5565

**Published:** 2025-06-12

**Authors:** Xinrong Li, Wenwen Si, Zhan Li, Ye Tian, Xuelei Liu, Shanyu Ye, Zifeng Huang, Yichun Ji, Caiping Zhao, Xiaoqian Hao, Dongfeng Chen, Meiling Zhu

Int J Mol Med 47: 61, 2021; DOI: 10.3892/ijmm.2021.4894

Subsequently to the publication of the above paper, an interested reader drew to the authors' attention that, for the JC-1 staining experiments shown in Fig. 5F on p. 9, the 'mimic NC' and 'inhibitor' panels appeared to show the same cells, even though the green/red ratio was different comparing between the two data panels. In addition, two further instances of duplicated data panels were identified in Figs. S2 and S3, such that data that were allegedly obtained under different experimental conditions appeared to have been derived from the same original sources.

After having asked the authors to explain the errors that had occurred in assembling these figures, they responded to explain that the error in Fig. 5F resulted from the incorrect selection of source images for the channels during the image merging process, whereas the errors in Figs. S2 and S3 occurred due to mistakes made in the naming and management of image files during storage. This led to the unintentional use of incorrect images during the process of figure assembly. Moreover, the authors were able to present to the Editorial Office the original data from the JC-1 staining experiments belonging to Fig. 5F. The Editor of *International Journal of Molecular Medicine* has agreed that a corrigendum may be published to account for the errors made in assembling Figs. 5, S2 and S3, and the corrected versions of these figures are shown on the next page. All the authors agree with the publication of this corrigendum, and are thankful to the Editor for giving them the opportunity to present this; moreover, the Editor and the authors apologize to the readership for any inconvenience caused.

## Figures and Tables

**Figure 5:**
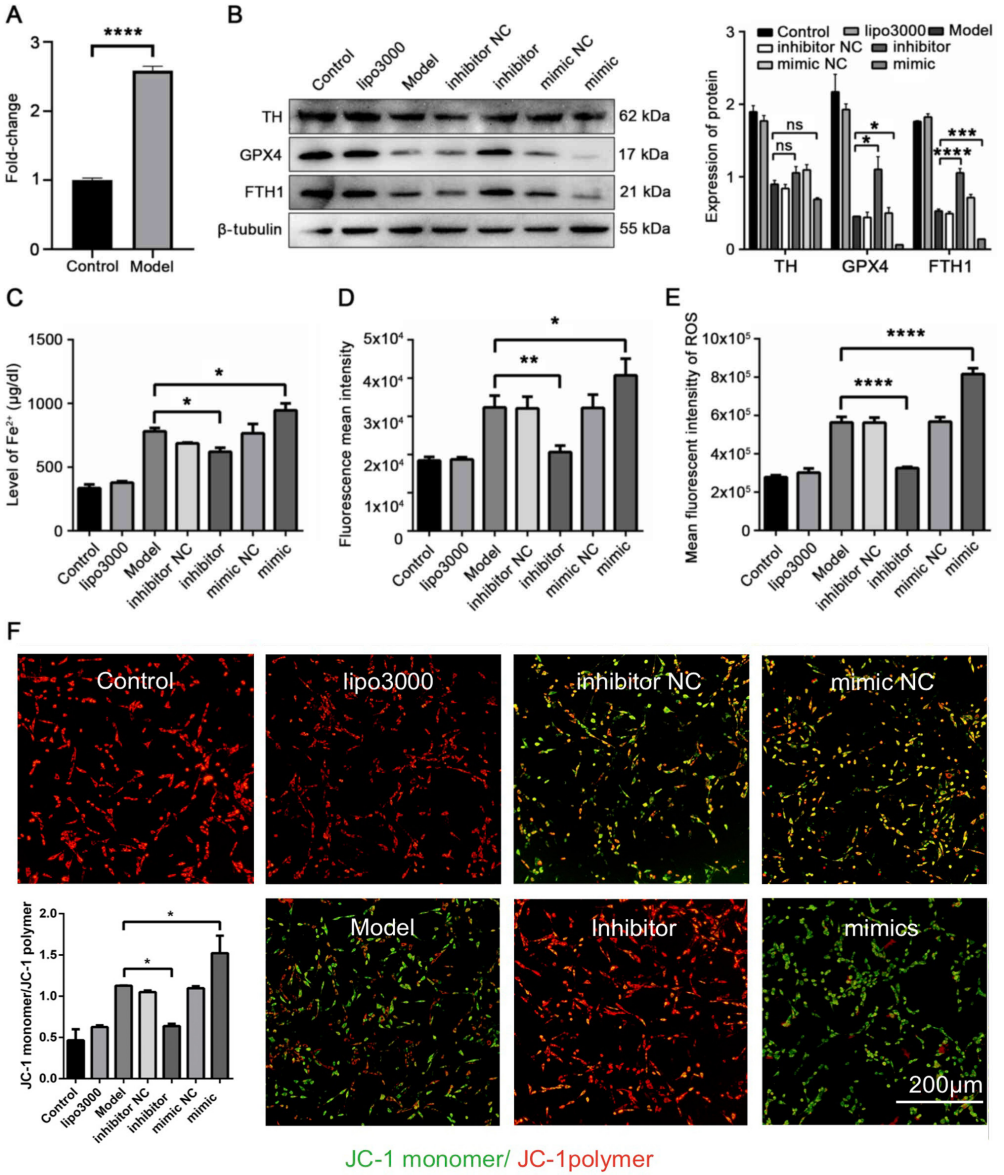
miR-335 promotes ferroptosis and reduces the expression of TH, GPX4 and FTH1 in 6-OHDA-stimulated cells. (A) Expression of miR-335 in 6-OHDA induced cells was markedly increased. (B) Expression of TH, GPX4 and FTH1 in PD cells was reduced by miR-335 mimic. (C) miR-335 increased the iron concentration in 6-OHDA-stimulated cells. (D) miR-335 increased lipid peroxidation in 6-OHDA-stimulated cells. (E) miR-335 significantly increased ROS levels in 6-OHDA-stimulated cells. (F) miR-335 downregulated MMP in 6-OHDA-stimulated cells. ^*^P<0.05, ^**^P<0.01, ^***^P<0.001 and ^****^P<0.0001 vs. model group; ns, not significant. Scale bar, 200 *µ*m. Parkinson's disease; TH, tyrosine hydroxylase; GPX4, glutathione peroxidase 4; FTH1, ferritin heavy chain 1; 6-OHDA, 6-hydroxydopamine.

**Figure S2:**
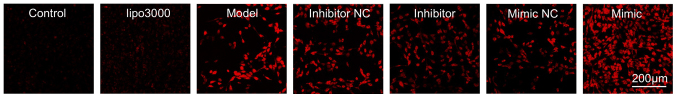
miR-355 further leads to the accumulation of ferrous ions in 6-OHDA-stimulated cells. Ferrous ion staining (red) was performed using the FeRhoNox-1 fluorescence imaging probe. 6-OHPA, 6-hydroxydopamine.

**Figure S3:**
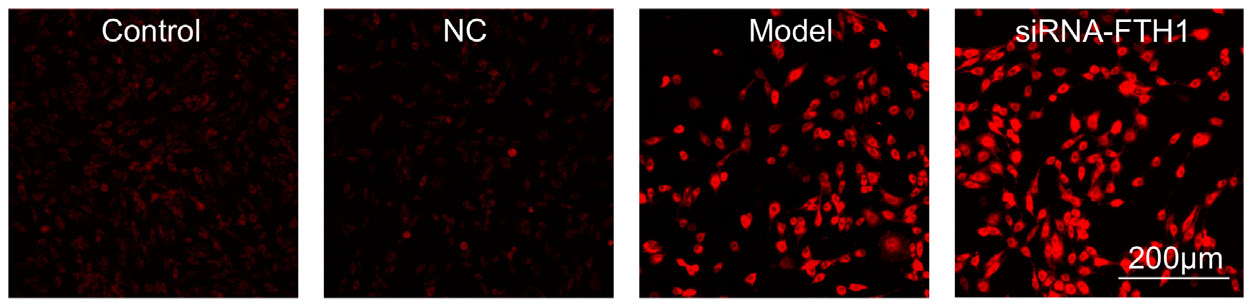
siRNA-FTH1 increases ferrous ion accumulation in 6-OHDA-stimulated cells. A FeRhoNox-1 fluorescence imaging probe was used for ferrous ion imaging of model cells and 6-OHDA-induced cells transfected with siRNA-FTH1. FTH1, ferritin heavy chain 1; 6-OHPA, 6-hydroxydopamine.

